# Effects of pneumatic tube systems on next-generation viscoelastic coagulation test devices in septic patients and healthy individuals: Results of the randomized controlled VETaPT trial

**DOI:** 10.1038/s41598-026-54938-7

**Published:** 2026-05-28

**Authors:** Martin Mirus, Erik Buehrer, Oliver Tiebel, Christian Schnabel, Jan Beyer-Westendorf, Thea Koch, Lars Thielecke, Peter Markus Spieth, Lars Heubner

**Affiliations:** 1 Department of Anaesthesiology and Intensive Care Medicine, Faculty of Medicine, University Hospital Carl Gustav Carus, TUD Dresden University of Technology, Fetscherstrasse 74, Dresden, 01307 Germany; 2 Institute of Clinical Chemistry, Faculty of Medicine, University Hospital Carl Gustav Carus, TUD Dresden University of Technology, Fetscherstrasse 74, Dresden, 01307 Germany; 3 Division of Haematology and Haemostasis, Department of Medicine I Thrombosis Research, Faculty of Medicine, University Hospital Carl Gustav Carus, TUD Dresden University of Technology, Fetscherstrasse 74, Dresden, 01307 Germany; 4 Institute for Medical Informatics and Biometry, Faculty of Medicine Carl Gustav Carus, TUD Dresden University of Technology, Fetscherstrasse 74, Dresden, 01307 Germany

**Keywords:** Coagulation, Platelet Function, Pneumatic Tube System, Point-of-Care, Sepsis, Viscoelastometry, Biomarkers, Diseases, Health care, Medical research

## Abstract

Rapid coagulation assessment is essential in critical care to enable timely correction of coagulopathy. Viscoelastic testing (VET) supports this goal but may be affected by mechanical stress during transport by pneumatic tube systems (PTS). As PTS are widely used to expedite sample delivery, evaluating the robustness of next-generation VET and platelet function assays under these conditions is crucial for reliable, time-sensitive diagnostics in intensive care. This study investigated the impact of PTS transport on VET and platelet function testing in healthy individuals and septic patients, including quantitative analysis of acceleration forces. This randomized trial applied a non-systematic sample-level allocation of paired blood samples from 46 healthy volunteers and 45 septic patients to manual and PTS transport. Acceleration was quantified via three-axis accelerometry. Samples were analyzed using ClotPro, ROTEM, TEG PlateletMapping, and Multiplate. Primary objective was the difference in test results following both transport modes. Analyses were performed on paired datasets (manual vs. PTS) per participant and assay. As pre-specified in the protocol, logistic regression modeled the probability of a clinically relevant EX-test clotting time (CT) change (≥ 10 s) within each cohort. Given the absence of associations, secondary equivalence analyses (TOST [two one-sided tests] and bootstrap) assessed whether observed effects were within pre-specified bounds. Neither logistic regression nor correlation analysis indicated an effect of mechanical stress on variable changes (all ρ < 0.5; *p* > 0.01). Across platforms, most viscoelastic and platelet function variables remained within predefined equivalence margins after PTS transport. Exceptions were TEG PlateletMapping HKH-R and, by bootstrap, ADP/AA-inhibition. In healthy volunteers, equivalence was confirmed for all variables (TOST *p* < 0.001). In septic patients, minor shifts remained within clinically acceptable limits, with equivalence confirmed for ClotPro IN-test CT (± 16s, p_lower_ = 0.036; p_upper_ < 0.001), EX-test MCF (± 2 mm, both *p* < 0.001), ROTEM INTEM CT (± 16s, both *p* < 0.001), Multiplate TRAP (± 10U, p_lower_ = 0.001; p_upper_ < 0.001), and TEG PlateletMapping ADP/AA inhibition (± 5%, both *p* < 0.05). Most next-generation viscoelastic and platelet assays are robust to PTS-induced stress. Coagulation diagnostics can include PTS transport without compromising validity. Only selected TEG PlateletMapping variables exhibited variability, indicating limited robustness. Trial registration: The study is retrospectively registered with the German Clinical Trials Register (DRKS00036231; https://drks.de/search/de/trial/DRKS00036231/details on 20.02.2025).

**Supplementary Information:**

The online version contains supplementary material available at 10.1038/s41598-026-54938-7.

## Introduction

Rapid coagulation assessment is crucial in acute hemorrhage, where timely correction prevents hypoperfusion and circulatory collapse. Point-of-care (POC) viscoelastic testing (VET) enables faster and more comprehensive coagulation profiling than standard laboratory tests (SLTs) such as prothrombin time, activated partial thromboplastin time (aPTT), platelet count, or fibrinogen^1–7^. Unlike conventional assays, VET provides real-time insights into clot formation, firmness, and fibrinolysis – key variables in critically ill patients with complex coagulopathies^8,9^. These insights support individualized hemostatic therapy and may reduce unnecessary transfusions^10^. Recent advances have expanded VET platforms^6,11,12^. However, VET results remain sensitive to preanalytical influences, particularly sample transport. Pneumatic tube systems (PTS), widely used to expedite delivery, expose samples to mechanical stress that may alter coagulation variables^13–17^. Previous studies show inconsistent findings, often lacking quantitative force measurement and relying on older devices or healthy subject, limiting clinical relevance^8,9,14–18^. The Viscoelastic Testing after Pneumatic Tube Transport (VETaPT) trial quantifies PTS-induced acceleration and evaluates its impact on next-generation VET and platelet function testing in healthy volunteers and septic patients. We hypothesize that PTS transport-related mechanical stress causes clinically relevant test deviations, potentially more pronounced in sepsis.

Primary objective is to determine whether PTS transport significantly affects viscoelastic and platelet function test results in septic patients compared with healthy controls. Secondary objectives include quantifying acceleration forces, assessing their correlation with test deviations, and identifying a clinically relevant acceleration threshold, facilitating broader applicability across PTS systems.

## Methods

This prospective, randomized clinical study with non-systematic sample-level allocation was reviewed and received a favorable opinion from the Ethics Committee of the Technical University Dresden, Germany (BO-EK-12012024_1), conducted in accordance with the Declaration of Helsinki, registered in the German Clinical Trials Register on February 20, 2025 (DRKS00036231, PI: Martin Mirus)^19^, and carried out at the Department of Anesthesiology and Intensive Care Medicine, University Hospital Dresden, Germany. All participants or their legal representatives provided written informed consent prior to enrollment. The methods section summarizes the study protocol and is based on the Consort Statement^20^. The detailed study protocol was published previously^21^.

### Study design

Paired blood samples from both healthy volunteers and critically ill patients with confirmed sepsis were collected, with each subject serving as their own control. Following collection, samples were distributed into identical, visually indistinguishable tubes and randomly assigned to either manual or PTS transport. This procedure resulted in a non-systematic allocation at the sample level, without the use of a predefined randomization sequence or centralized software-based allocation. Each participant therefore contributed paired measurements for each assay, forming the basis of the dataset-level analyses. The PTS used in this study was an Aerocom AC4000 with a tube diameter 160 mm (Aerocom, Schwäbisch-Gmünd, Germany) with a transport distance of approximately 500 m. The median transport time was 5 min (interquartile range 3–7 min). The system included approximately 40 bends. Transport velocities are set at 2 m/s for standard clinical samples and 6–7 m/s for non-clinical transports by institutional default. Both velocity settings were evaluated, along with transport configurations using either carrier bags or fixed tube holders. Sample fixation (bag vs. insert) was predefined and is described in detail in the methods publication^21^.

Acceleration forces during PTS transport were continuously recorded via a three-axis accelerometer. Samples were analyzed via standard laboratory tests and four POC devices for coagulation assessment. Detailed study design was published previously^21^. ROTEM, standard laboratory testing, and Multiplate analyses were performed by laboratory personnel who were unaware of transport allocation. ClotPro and TEG PlateletMapping measurements were performed by the investigator, who was aware of sample allocation at the time of analysis.

### Participants

Inclusion criteria for healthy volunteers were age ≥ 18 years, absence of known coagulation disorders, and no intake of anticoagulant or antiplatelet medication within the previous seven days. Inclusion criteria for septic patients were age ≥ 18 years, diagnosis of sepsis or septic shock within the preceding 48 h according to SEPSIS-3 criteria^22^, Sequential Organ Failure Assessment (SOFA) score of ≥ 9, current anticoagulation therapy with unfractionated heparin (UFH), low-molecular-weight heparin (LMWH), or argatroban at any dosage. Exclusion criteria for both groups included known hereditary coagulation disorders and withdrawal of informed consent.

### Intervention and measurements

#### Point-of-care devices

Samples were analyzed via following POC platforms: ClotPro (Haemonetics, United States), ROTEM sigma (Werfen TEM Innovations, Munich), TEG6s with TEG PlateletMapping (Haemonetics, United States), and Multiplate (Roche Diagnostics, Basel, Switzerland). The viscoelastic devices measured key variables of coagulation, including clot initiation (clotting time [CT] or reaction time [R]), clot propagation (clot formation time [CFT]), clot strength (maximum clot firmness [MCF] or maximum amplitude [MA]), and clot stability (lysis time). TEG PlateletMapping and Multiplate aggregometry further assessed platelet inhibition using standard tests triggered by adenosine diphosphate (ADP), arachidonic acid (AA, ASPI test), and thrombin receptor–activating peptide (TRAP). Draw-to-analysis times were recorded for ClotPro, ROTEM, and TEG. For Multiplate, a standardized resting period prior to analysis was applied according to manufacturer recommendations (approximately 30 min after sampling). Samples from both transport conditions were processed under identical laboratory conditions. Temperature during transport was not actively controlled and followed routine clinical conditions. The study protocol provides further descriptions of the devices and tests used in this study^21^.

### Quantification of acceleration forces

During transport, acceleration was continuously recorded via a three-axis accelerometer (MSR 145, MSR Electronics, Switzerland). Acceleration, including gravitational pull, was measured as multiples of gravitational force (g) per axis at 50 Hz and vector magnitude calculated due to the changing orientation of the accelerometer. Quantification was done via an area under the curve (AUC) approach and additionally normalized for sampling rate^21^. AUC calculation and threshold choice followed Streichert at al^23^. and is more extensively described in the study protocol of this study^21^. To systematically introduce controlled variations in acceleration forces and investigate thresholds at which PTS-induced stress affects coagulation test results, predefined transport conditions were randomly applied^1^: high versus low transport speed, and^2^ transport of samples in a small transport bag versus a fixed-position insert. This approach aimed to increase variability in the AUC, serving as a composite measure of the total acceleration forces applied during transport^21^.

### Data handling

Data were pseudonymized and managed via electronic systems in compliance with institutional data protection policies. Clinical and demographic data were recorded from hospital documentation systems and direct surveys.

### Primary and secondary endpoints

The primary endpoint was the difference in coagulation and platelet function variables between PTS and manually transported samples. Secondary endpoints included the correlation between acceleration forces, quantified as AUC, and changes in coagulation test results, the identification of a clinically relevant acceleration threshold beyond which test results are significantly affected, and the comparison of susceptibility to acceleration-induced effects between critically ill septic patients and healthy individuals.

### Sample size calculation

The detailed sample size calculation has been published in the study protocol^21^. Briefly, a ≥ 10 s difference in ClotPro Ex-test CT between manual and PTS-transported samples defined a binary clinically relevant outcome. To model the probability of observing such a change, a logistic regression approach was chosen. The dependent variable was the binary outcome, and the primary independent variable was the AUC of acceleration forces during transport. On the basis of logistic regression modeling, the required sample size was calculated via G*Power 3.1 (α = 0.05, power = 0.80)^24^. A total of 83 paired comparisons were needed. Assuming a 10% dropout rate, a final sample size of 92 participants (46 per group) was planned^21^.

### Statistical analyses

The unit of analysis for transport comparisons was the paired dataset, defined as matched manual and PTS measurements obtained from the same participant for a given assay. Thus, each participant could contribute multiple paired datasets across different devices and test parameters. The term “dataset” does not refer to individual blood tubes or participants, but to assay-specific paired comparisons. Baseline differences between variables were assessed using t-tests or Wilcoxon rank-sum tests, depending on the distribution of the data, as determined by the Shapiro-Wilk test. Binary influence of acceleration forces on test outcomes was analyzed via logistic regression as described above for sample size calculation. A p-value ≤ 0.05 was considered statistically significant. The initial analytical approach using logistic regression was extended to include equivalence testing between transport conditions, as logistic regression proved unsuitable given the data distribution and the absence of a detectable association. Two statistical approaches were used to determine whether the observed effects remained within predefined equivalence margins. The equivalence margins were defined prior to analysis and set at the level of the coefficient of variation (CV) reported by the respective device manufacturers. For technically similar systems (ClotPro and ROTEM), equal margins were chosen. The first approach was the two one-sided test procedure (TOST^25^, as outlined previously^26,27^. TOST does not test whether an effect differs from zero but rather whether it lies within specified upper and lower bounds. If both one-sided null hypotheses – the effect exceeds the upper bound or falls below the lower bound – can be rejected at α < 0.05, the observed effect is considered statistically equivalent. The TOST procedure provides two one-sided p-values, corresponding to the upper (p_upper_) and lower (p_lower_) equivalence bounds. Owing to non-normal data distribution, we applied two one-sided Wilcoxon signed-rank tests as a non-parametric implementation of TOST. Results were calculated in MATLAB and cross-validated via the R-package provided by Caldwell and colleagues^27^. The second approach used a bootstrap-based method. Here, 10,000 resamples were drawn to estimate confidence intervals for the median difference between transport methods. These intervals were compared to the predefined equivalence margins. Normalizing effect sizes relative to these margins enables a standardized comparison across variables. Equivalence testing was considered the primary framework. Conventional testing (Wilcoxon tests) was performed for descriptive purposes only and not used for confirmatory inference. Missing data were handled by device-specific complete-case analysis. The analytical unit was the paired dataset (manual vs. PTS) per participant and assay. Accordingly, participants were included in a given analysis only if both paired measurements were available for the respective device and parameter. Missingness occurred primarily at the device level and was mainly attributable to technical or logistical unavailability of individual measurements rather than participant exclusion. As the underlying missing-data mechanism was not formally tested, complete-case analysis was applied as a pragmatic approach; its limitations are acknowledged. Unless otherwise specified, values in the tables are reported as medians with the first and third quartiles (Q25 - Q75). All statistical analyses were performed via MATLAB (The MathWorks Inc., 2024) and RStudio (Posit Software, PBC, Boston, USA, version 2025.05.0 + 496).

## Results

### Study participants

**Fig. 1:**
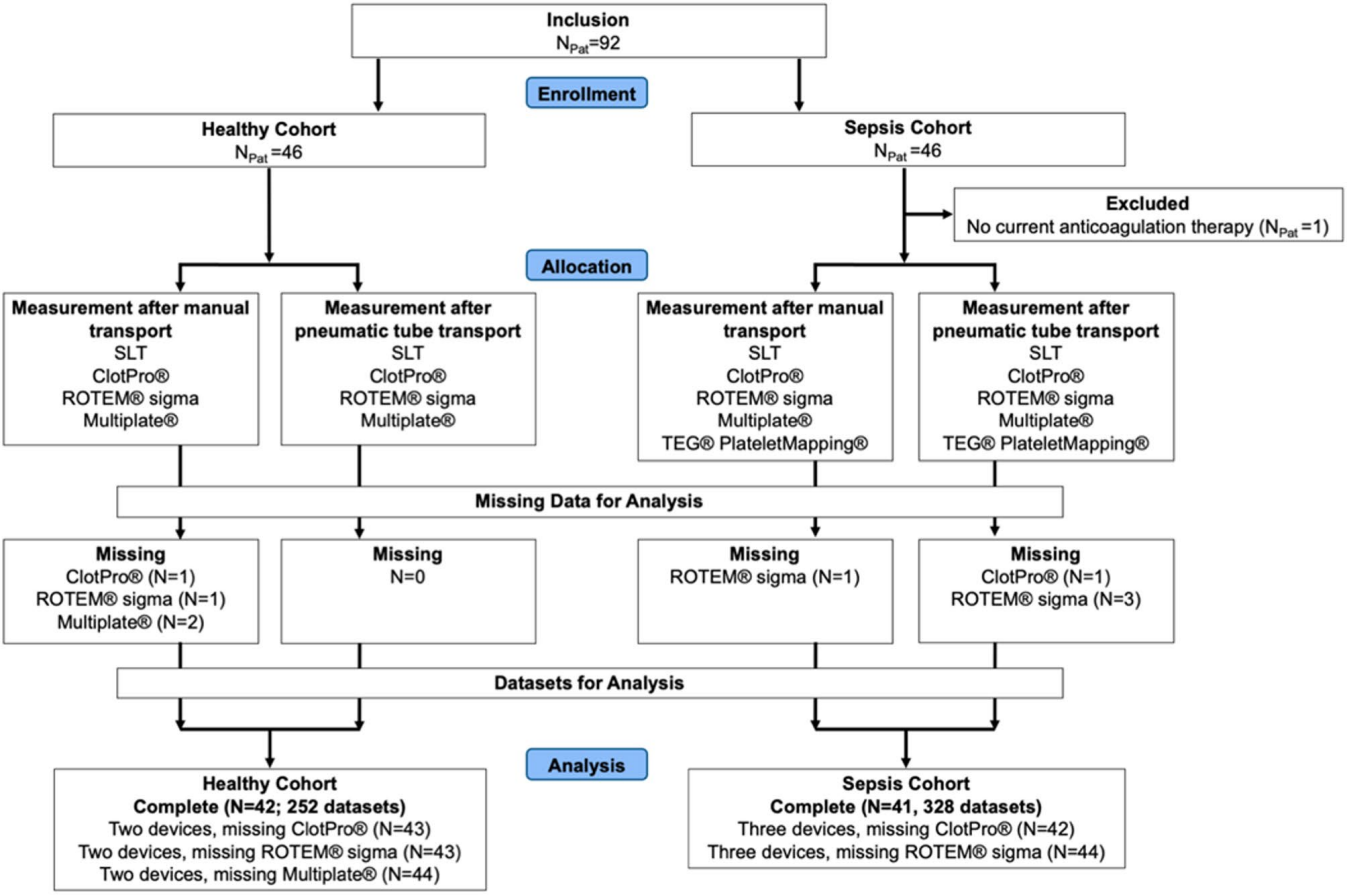
CONSORT diagram illustrating cohort allocation, device availability, and data completeness.

The study CONSORT diagram is shown in Fig. 1. A total of 92 individuals were enrolled, comprising 46 healthy volunteers and 46 septic patients. One septic patient was excluded because no current anticoagulation therapy was present, leaving 45 septic patients for analysis.

Median draw-to-analysis times were comparable between transport modalities: ClotPro 30 (21–38) min vs. 28 (20–35) min, ROTEM 57 (41–79) min vs. 58 (41–83) min, and TEG 62 (29–95) min vs. 55 (35–99) min for manual and PTS transport, respectively. For transport comparisons, the relevant analytical unit was the paired dataset per participant and assay. Because missingness occurred at the device level, the number of analyzable paired datasets differed between platforms. In the healthy cohort, 42 participants contributed complete datasets across all devices (252 paired datasets), whereas in the septic cohort, 41 participants contributed complete datasets (328 paired datasets). Participants with missing measurements on individual devices were still included in analyses of other assays where paired data were available. Participant recruitment took place between June 2024 and April 2025. The healthy cohort comprised 46 volunteers with a median age of 27 (25–35) years, 41% of whom were female. The sepsis cohort included 45 patients with a median age of 64 (55–74) years and a body weight of 80 (70–96) kg; 26% were female. At the time of sampling, median SOFA, SAPS II, and APACHE II scores were 10.0 (9.0–12.0), 37 (33–46), and 20 (14–23), respectively. SOFA scores are shown in Figure S1A. Laboratory values revealed a median leukocyte count of 14.7 (9.5–22.4) × 10⁹/L and a platelet count of 184 (120–273) × 10⁹/L. The C-reactive protein (CRP) concentration was 156.8 (117.0–276.5) mg/L, and procalcitonin (PCT) concentration was 6.5 (1.8–21.8) ng/mL. Suspected sepsis foci and pathogens detected in positive blood cultures are shown in Figure S2AB. Drugs administered for thromboprophylaxis, anticoagulation, or platelet inhibition in the septic cohort are depicted in Figure S1B. UFH was administered in 24 patients at a median dose of 600IU/h (400–1250). LMWH included tinzaparin in 16 patients (5250IU/day [4500–9500]) and certoparin in one patient (3000IU/day). Argatroban was used in four patients at a median dose of 0.15 µg/kg/min (0.07–0.54).

Antiplatelet therapy was present in a subset of patients. Acetylsalicylic acid (ASA) was administered in 10 patients at a dose of 100 mg/day, and ticagrelor in one patient (75 mg/day). Dual antiplatelet therapy consisted of ASA plus clopidogrel in two patients (100 mg + 75 mg/day) and ASA plus ticagrelor in two patients (100 mg + 90 mg/day).

CONSORT diagram summarizing the inclusion, allocation, and data availability of the studies participants in the VETaPT trial. A total of 92 subjects were enrolled, comprising 46 healthy volunteers and 46 critically ill septic patients. One septic patient was excluded due to absence of current anticoagulation therapy, resulting in 45 septic patients for analysis. For transport comparisons, the analytical unit was the paired dataset, defined as matched manual and PTS measurements from the same participant for a given assay. Because missingness occurred at the device level, the number of analyzable datasets differs across platforms. The “N” reported under missing data refers to the number of missing paired datasets for the respective device, i.e., cases in which at least one of the two required measurements (manual or PTS) was unavailable for that device. The term “complete” refers to participants with available paired measurements across all devices, whereas device-specific missingness indicates absence of paired data for individual assays. Blood samples were analyzed using ClotPro, ROTEM sigma, Multiplate, and TEG PlateletMapping following either manual or pneumatic tube transport. The figure displays the number of complete datasets per device and transport modality as well as the number of missing measurements. One patient was excluded due to absence of current anticoagulation therapy. N_Pat_, number of participants; N, number of individuals with paired comparisons for one or more devices; SLT, standard laboratory testing; PTS, pneumatic tube system.

### Transport-related differences in coagulation and platelet function variables

**Fig. 2:**
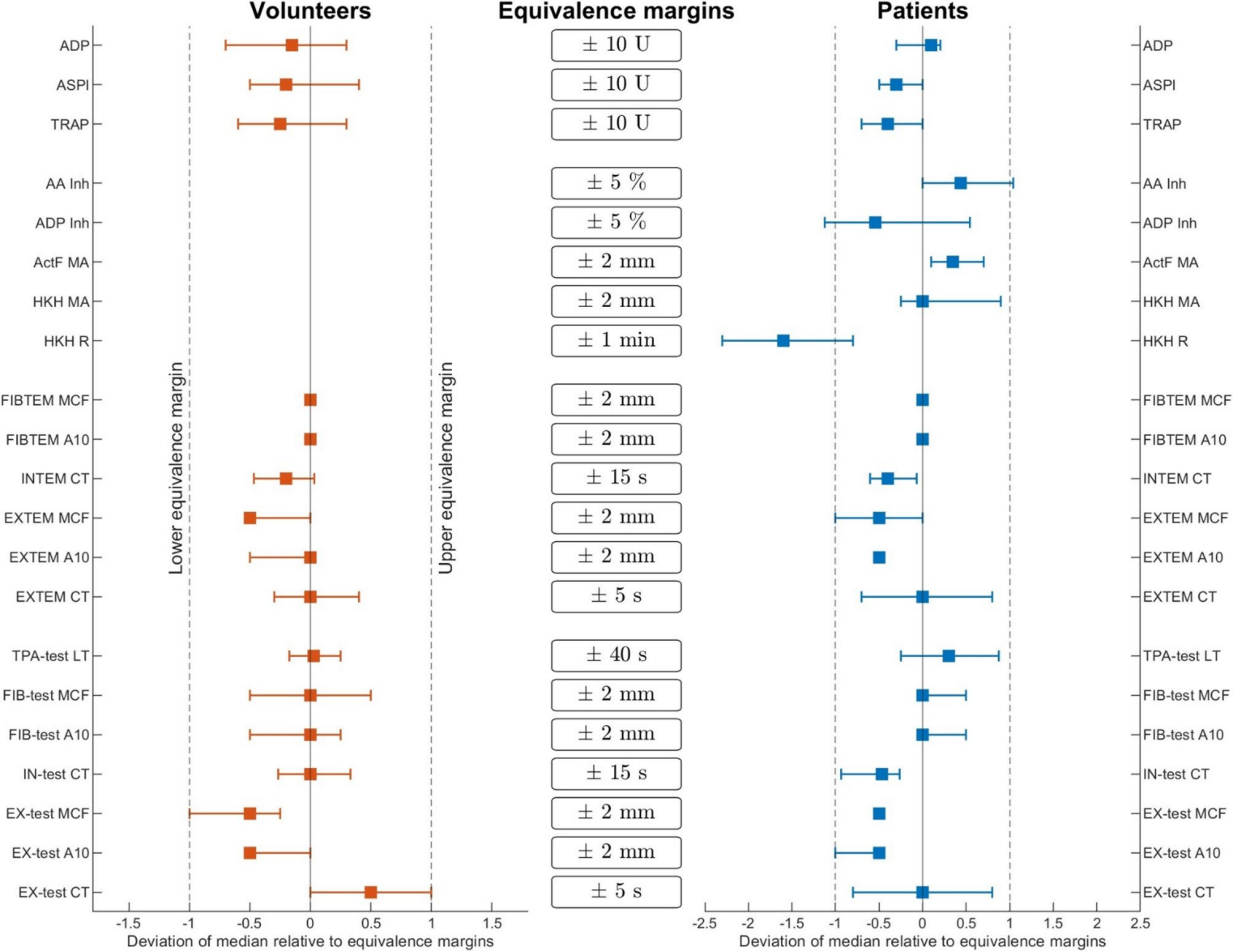
Median differences in viscoelastic and platelet function variables following both transport methods. Equivalence analysis comparing PTS and manual transport. X-axis represents deviation of median difference between both transport methods expressed relative to equivalence margins, based on coefficients of variation provided by manufacturers. Results separately for healthy volunteers (left) and septic patients (right). Horizontal lines represent 2.5% and 97.5% quantiles, derived via bootstrap method on basis of repeated random sampling from this respective cohort. Equivalence is assumed if interval lies within the region defined by equivalence margins (vertical lines). Variables outside this range are considered nonequivalent. Values normalized to the respective equivalence thresholds. Confidence intervals for HKH R and ADP MA exceed predefined equivalence margins. For HKH R, even median of all measurements lies outside the selected threshold. The absence of whiskers indicates identical medians across bootstrap replicates within the respective cohort. ADP, adenosine diphosphate; ASPI, arachidonic acid–induced aggregation; TRAP, thrombin receptor–activating peptide; AA/ADP Inh, % inhibition; ActF, activated functional fibrinogen; HKH, heparinized kaolin-activated heparinase; MA, maximum amplitude; R, reaction time; CT, clotting time; A10, amplitude at 10 min; MCF, maximum clot firmness; EX-test, extrinsic activation; IN-test, intrinsic activation; FIB-test, fibrinogen contribution; PTS, pneumatic tube system.

In healthy volunteers, statistical equivalence was confirmed across all variables via both approaches. TOST yielded p-values < 0.001 for all comparisons (Table 1), and bootstrap-derived confidence intervals for median differences remained entirely within the predefined equivalence margins specified by the respective device manufacturers (Fig. 2).

In septic patients, statistical equivalence was confirmed for most variables via both approaches. Minor deviations remained within acceptable limits, with TOST confirming equivalence for ClotPro, ROTEM, Multiplate, and ADP/AA inhibition in TEG PlateletMapping (Table 2). Deviations from statistical equivalence in septic patients were observed only with the bootstrap-based method for TEG PlateletMapping for the HKH-R and with the ADP/AA-inhibition of platelet activation (Fig. 2).

The five transport modes led to distinct AUC profiles: manual transport showed the lowest exposure (5 [1–12] g·s), followed by PTS with slow speed and bag (119 [99–132] g·s), slow speed with insert (148 [139–158] g·s), fast speed with bag (225 [191–250] g·s), and fast speed with insert (271 [262–278] g·s); averaged PTS transports yielded 185 [138–263] g·s. Table S1 provides the distribution of acceleration magnitudes across defined bins for these transport modes.

**Table 1:** Results of standard laboratory, viscoelastic and platelet tests for healthy volunteers.

		Healthy Volunteers						
		Manual	PTS	TOST			Difference	
				∆	*p*_lower_	*p*_upper_	Median	*p*-value
***Conventional Lab***	***Reference***							
Quick [%]	70–120	90 (86–96)	92 (86–98)	± 10	< 0.001	< 0.001	0 (−1–1)	0.581
INR [1]	0.90–1.20	1.07 (1.03–1.11)	1.06 (1.01–1.11)	± 0.1	< 0.001	< 0.001	0.00 (−0.01–0.01)	0.967
aPTT [s]	24–36	30 (28–31)	30 (28–31)	± 3	< 0.001	< 0.001	0 (0–0)	0.491
Fibrinogen [g/L]	2.0–4.0	2.7 (2.3–3.0)	2.8 (2.4–3.0)	± 0.5	< 0.001	< 0.001	0.0 (0.0–0.1)	0.234
*ClotPro*	*Reference*							
EX-test CT [s]	38–65	51 (45–56)	54 (50–59)	± 5	< 0.001	0.034	2 (−2–9)	0.053
EX-test A10 [mm]	47–64	54 (50–56)	52 (51–55)	± 2	< 0.001	< 0.001	−1 (−1–0)	**0.005**
EX-test MCF [mm]	53–68	58 (55–60)	57 (55–59)	± 2	< 0.001	< 0.001	−1 (−2–0)	**< 0.001**
IN-test CT [s]	139–187	152 (142–158)	151 (142–160)	± 16	< 0.001	< 0.001	0 (−6–8)	0.887
FIB-test A10 [mm]	7–23	12 (9–15)	11 (9–15)	± 2	< 0.001	< 0.001	0 (−1–1)	0.371
FIB-test MCF [mm]	9–27	14 (10–17)	14 (11–17)	± 2	< 0.001	< 0.001	0 (−1–1)	0.761
TPA-test LT [s]	151–411	165 (146–184)	167 (145–197)	40	< 0.001	< 0.001	1 (−12–16)	0.483
*ROTEM*	*Reference*							
EXTEM CT [s]	50–80	64 (58–68)	64 (59–69)	± 5	< 0.001	< 0.001	0 (−3–5)	0.659
EXTEM A10 [mm]	43–63	53 (50–56)	53 (49–57)	± 2	< 0.001	< 0.001	0 (−2–1)	0.106
EXTEM MCF [mm]	55–72	61 (58–64)	61 (58–64)	± 2	< 0.001	< 0.001	−1 (−2–0)	**0.017**
INTEM CT [s]	161–204	194 (186–201)	189 (180–198)	± 16	< 0.001	< 0.001	−3 (−10–2)	**0.013**
FIBTEM A10 [mm]	6–21	9 (7–11)	8 (7–11)	± 2	< 0.001	< 0.001	0 (−1–0)	0.181
FIBTEM MCF [mm]	6–21	10 (7–12)	10 (7–12)	± 2	< 0.001	< 0.001	0 (−1–1)	0.502
*Multiplate*	*Reference*							
TRAP AUC [U]	70–128	126 (110–139)	123 (111–146)	± 10	< 0.001	< 0.001	−2 (−10–8)	0.643
ASPI AUC [U]	71–115	104 (94–115)	106 (93–116)	± 10	< 0.001	< 0.001	−2 (−8–8)	0.955
ADP AUC [U]	57–113	60 (46–80)	62 (44–77)	± 10	< 0.001	< 0.001	−2 (−9–11)	0.866

Table 1: Comparison of conventional laboratory variables, VET results, and platelet function test results in healthy volunteers after manual versus PTS transport. Equivalence was assessed using the TOST procedure with predefined manufacturer-based equivalence margins (∆), p_lower_ and p_upper_ denote the p-values of the lower and upper one-sided test of the TOST, respectively. The listed reference values are based on the specifications provided by the laboratory of University Hospital Dresden (for conventional coagulation tests) and according to the respective manufacturers for the point-of-care devices (ClotPro, ROTEM, Multiplate). Data are reported as medians with the 25th and 75th percentiles; p-values correspond to the Wilcoxon signed-rank test. VET, viscoelastic testing; PTS, pneumatic tube system; TOST, two one-sided test; INR, international normalized ratio; aPTT, activated partial thromboplastin time; CT, clotting time; A10, clot amplitude after 10 min; MCF, maximum clot firmness; LT, lysis time; AUC, area under the curve; TRAP, thrombin receptor-activating peptide; ASPI, arachidonic acid stimulation test; ADP, adenosine diphosphate stimulation test; EX-test, extrinsic pathway test (ClotPro); IN-test, intrinsic pathway test (ClotPro); FIB-test, fibrinogen test (ClotPro); TPA-test, tissue plasminogen activator test (ClotPro); EXTEM, extrinsic thromboelastometry (ROTEM); INTEM, intrinsic thromboelastometry (ROTEM); FIBTEM, fibrin-based thromboelastometry (ROTEM).

**Table 2:** Results of standard laboratory, viscoelastic and platelet tests for septic patients.

		Septic Patients						
		Manual	PTS	TOST			Difference	
				∆	*p*_lower_	*p*_upper_	Median	*p*-value
***Conventional Lab***	***Reference***							
Quick [%]	70–120	70 (58–84)	70 (58–84)	± 10	< 0.001	< 0.001	0 (−1–1)	0.65
INR [1] aPTT [s] Fibrinogen [g/L]	0.9–1.2	1.29 (1.13–1.49)	1.29 (1.12–1.48)	± 0.1	< 0.001	< 0.001	0 (−0.01–0.01)	0.849
	24–36	33 (29–45)	32 (28–44)	± 3	< 0.001	< 0.001	0 (−1–0)	**0.007**
	2.0–4.0	5.5 (4.1–6.4)	5.4 (3.6–6.4)	± 0.5	< 0.001	< 0.001	−0 (−0.1–0.1)	0.415
*ClotPro*	*Reference*							
EX-test CT [s]	38–65	59 (51–76)	60 (52–75)	± 5	0.005	< 0.001	0 (−7–7)	0.684
EX-test A10 [mm]	47–64	64 (56–67)	62 (54–66)	± 2	0.006	< 0.001	−1 (−2–0)	**< 0.001**
EX-test MCF [mm]	53–68	67 (61–69)	65 (59–68)	± 2	< 0.001	< 0.001	−1 (−2–0)	**< 0.001**
IN-test CT [s]	139–187	190 (161–267)	184 (157–240)	± 16	0.036	< 0.001	−7 (−18–1)	**< 0.001**
FIB-test A10 [mm]	7–23	29 (23–34)	29 (22–32)	± 2	< 0.001	< 0.001	0 (−1–1)	0.795
FIB-test MCF [mm]	9–27	32 (26–37)	33 (26–35)	± 2	< 0.001	< 0.001	0 (−1–1)	0.718
TPA-test LT [s]	151–411	386 (308–458)	363 (301–494)	40	< 0.001	0.043	12 (−16–50)	0.095
*ROTEM*	*Reference*							
EXTEM CT [s]	50–80	70 (62–88)	70 (62–90)	± 5	0.008	0.018	0 (−5–9)	0.717
EXTEM A10 [mm]	43–63	66 (58–70)	66 (56–70)	± 2	0.001	< 0.001	−1 (−2–0)	**0.003**
EXTEM MCF [mm]	55–72	72 (64–77)	72 (62–75)	± 2	< 0.001	< 0.001	−1 (−2–0)	**< 0.001**
INTEM CT [s]	161–204	238 (192–290)	231 (190–284)	± 16	< 0.001	< 0.001	−6 (−12–2)	**0.007**
FIBTEM A10 [mm]	6–21	26 (20–30)	26 (21–32)	± 2	< 0.001	< 0.001	0 (−1–0)	0.864
FIBTEM MCF [mm]	6–21	29 (22–33)	29 (22–34)	± 2	< 0.001	< 0.001	0 (−1–0)	0.064
*Multiplate*	*Reference*							
TRAP AUC [U]	70–128	58 (41–91)	58 (34–86)	± 10	0.001	< 0.001	−4 (−10–3)	**0.021**
ASPI AUC [U]	71–115	43 (21–79)	36 (20–79)	± 10	< 0.001	< 0.001	−3 (−9–3)	0.094
ADP AUC [U]	57–113	23 (18–37)	24 (16–38)	± 10	< 0.001	< 0.001	1 (−4–4)	0.743
*TEG Platelet Mapping*	*Reference*							
HKH R [min]	4.2–9.8	6 (5–8)	5 (4–6)	± 1	**0.991**	< 0.001	−2 (−3 - −1)	**< 0.001**
HKH MA [mm]	53.0–68.0	68 (63–71)	69 (63–72)	± 2	< 0.001	0.010	0 (−1–2)	0.241
ActF MA [mm]	2.0–19.0	27 (19–41)	29 (19–42)	± 2	< 0.001	0.037	1 (0–2)	**0.010**
ADP MA [mm]	45.0–69.0	57 (41–64)	58 (45–65)	± 3	< 0.001	**0.085**	1 (−3–5)	0.183
ADP Inh [%]	0–17	31 (13–51)	28 (18–43)	± 8	0.040	< 0.001	−3 (−10–7)	0.397
AA MA [mm]	51.0–71.0	67 (53–70)	65 (52–71)	± 3	0.003	0.002	−0 (−2–1)	0.44
AA Inh [%]	0–11	4 (0–22)	10 (2–26)	± 5	0.001	0.036	2 (−0–6)	0.104

Table 2: Comparison of conventional laboratory variables, VET results, and platelet function test results in septic patients after manual versus PTS transport. Equivalence was assessed using the TOST procedure with predefined manufacturer-based equivalence margins (∆), p_lower_ and p_upper_ denote the p-values of the lower and upper one-sided test of the TOST, respectively. The listed reference values are based on the specifications provided by the laboratory of University Hospital Dresden (for conventional coagulation tests) and according to the respective manufacturers for the point-of-care devices (ClotPro, ROTEM, Multiplate, TEG PlateletMapping). Data are reported as medians with the 25th and 75th percentiles; p-values correspond to the Wilcoxon signed-rank test. VET, viscoelastic testing; PTS, pneumatic tube system; TOST, two one-sided test; INR, international normalized ratio; aPTT, activated partial thromboplastin time; Quick, prothrombin time expressed as percentage; fibrinogen, fibrinogen concentration; CT, clotting time; A10, clot amplitude after 10 min; MCF, maximum clot firmness; LT, lysis time; AUC, area under the curve; TRAP, thrombin receptor-activating peptide; ASPI, arachidonic acid stimulation test; ADP, adenosine diphosphate stimulation test; EX-test, extrinsic pathway test (ClotPro); IN-test, intrinsic pathway test (ClotPro); FIB-test, fibrinogen test (ClotPro); TPA-test, tissue plasminogen activator test (ClotPro); EXTEM, extrinsic thromboelastometry (ROTEM); INTEM, intrinsic thromboelastometry (ROTEM); FIBTEM, fibrin-based thromboelastometry (ROTEM); TEG Platelet Mapping: HKH R, kaolin-activated heparinase reaction time; HKH MA, kaolin-activated heparinase maximum amplitude; ActF MA, activator F maximum amplitude; ADP MA, adenosine diphosphate maximum amplitude; ADP Inh, adenosine diphosphate inhibition; AA MA, arachidonic acid maximum amplitude; AA Inh, arachidonic acid inhibition.

### ClotPro

**Fig. 3:**
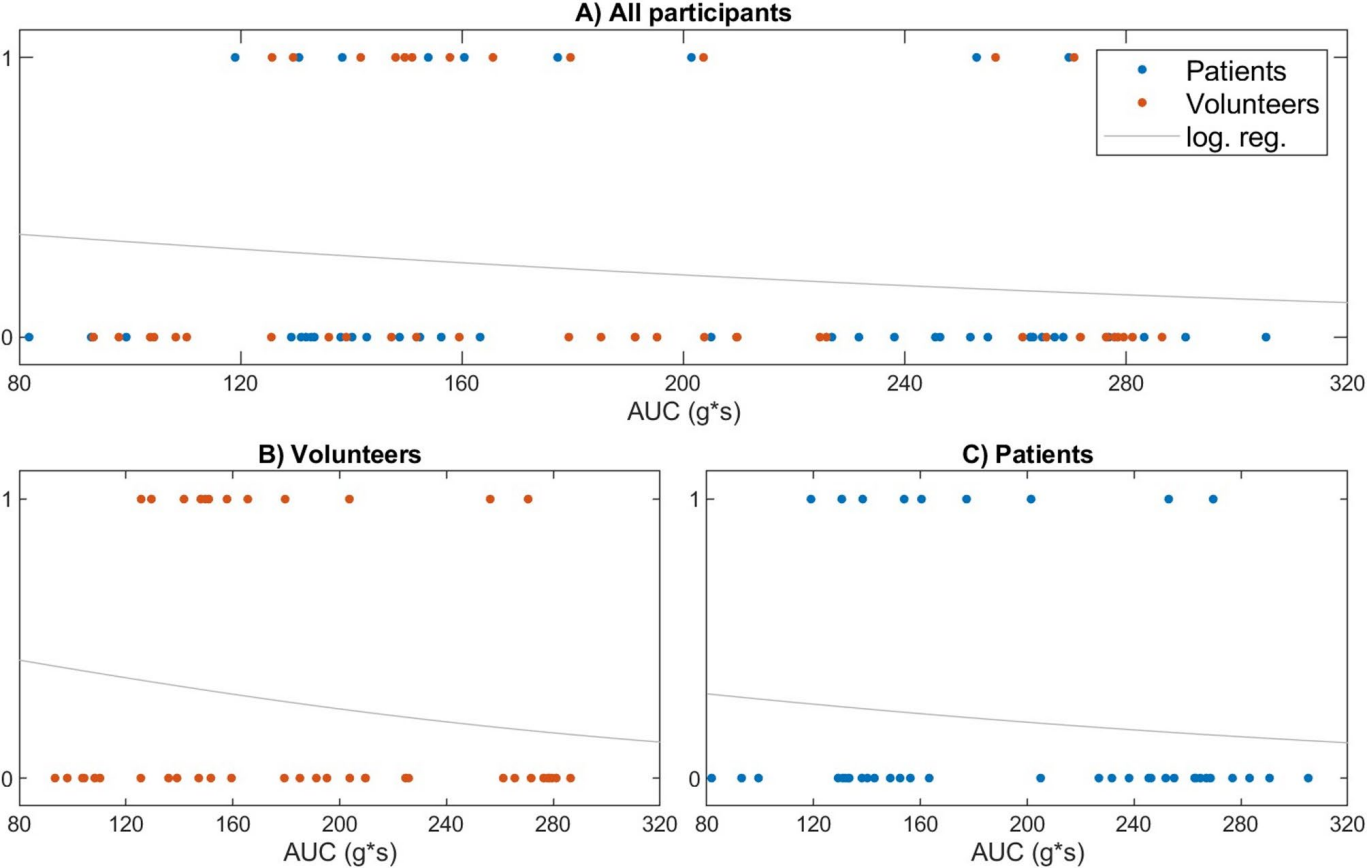
**A-C. **Logistic regression analysis for the association between the AUC and the probability of clinically relevant deviations in the viscoelastic test results. Logistic regression analysis evaluating the predictive value of transport-related acceleration forces (expressed as AUC) for clinically relevant deviations in viscoelastic coagulation test results. A: The upper panel A displays all the study participants (patients and healthy volunteers). B, C: The lower panels show subgroup analyses for volunteers (left, B) and patients (right, C). The data points represent individual paired measurements after pneumatic tube transport. The AUC values on the x-axis are plotted against the binary outcome (0 = no relevant deviation, 1 = clinically relevant deviation) on the y-axis. The regression line suggests no relevant associations in either subgroup. AUC, area under the curve.

In the EX-test, neither CT nor α-angle statistically significant differed after PTS transport in either cohort. MCF was reduced by 1 mm post transport in both groups. Equivalence testing demonstrated statistical equivalence for CT, A10, and MCF within the predefined equivalence margins in both cohorts. In the IN-test, CT remained unchanged after PTS transport in healthy volunteers. In septic patients, CT was shortened by 7 s. Equivalence testing confirmed statistical equivalence for healthy volunteers and septic patients at the predefined margin of ± 16 s. In the FIB-test, neither A10 nor MCF showed significant differences following PTS transport in either cohort. Equivalence testing confirmed statistical equivalence for both variables in both groups. In the TPA-test, lysis time did not differ significantly after PTS transport in either cohort. Equivalence testing confirmed statistical equivalence of the lysis time in both groups. The influence of PTS transport on CT in the EX-test was initially analyzed via logistic regression (Fig. 3A-C), as described above. The resulting regression curve demonstrated a negative slope. This trend was observed in the combined cohort and when healthy volunteers and septic patients were analyzed separately. The corresponding p-values calculated via chi-square test were 0.137 for the overall cohort, 0.237 for healthy volunteers, and 0.432 for septic patients.

### ROTEM

In the EXTEM test, CT did not differ significantly after PTS transport in either healthy volunteers or septic patients. MCF was reduced by 1 mm in both cohorts. Equivalence testing confirmed statistical equivalence for CT, A10, and MCF within the predefined equivalence margins in both groups. In the INTEM test, CT was shortened by 1 s in healthy volunteers and by 6 s in septic patients following PTS. In both cohorts, equivalence testing demonstrated statistical equivalence for CT within the specified equivalence margins.

In the FIBTEM test, A10 and MCF did not statistically significant differ after PTS transport in either cohort. Equivalence testing confirmed statistical equivalence for both variables within the predefined margins in both groups.

### Multiplate

In healthy volunteers, the TRAP, ASPI, and ADP test revealed no statistically significant differences following PTS transport (Table 1). In septic patients, the ASPI and ADP test results also remained unchanged, whereas the TRAP test results revealed a median decrease of 4 units (Table 2). Equivalence testing demonstrated statistical equivalence for TRAP, ASPI, and ADP tests in both cohorts within the predefined equivalence margins (Tables 1 and 2; Fig. 2).

### TEG platelet mapping

In septic patients, PTS transport resulted in a 2-minute shortening of the R value in the HKH test and a 1-mm increase in the MA of the activator F (ActF) test, the latter reflecting fibrinogen contribution. Equivalence testing via the TOST procedure demonstrated statistical inequivalence for the HKH R value (Table 2). Bootstrap-based analysis revealed that the 95% confidence intervals for HKH R, as well as ADP- and AA-induced platelet inhibition, exceeded the predefined equivalence margins. Notably, for the HKH R, even the median difference fell outside the accepted threshold (Fig. 2).

### Correlation between acceleration and test result changes after PTS transport

**Fig. 4:**
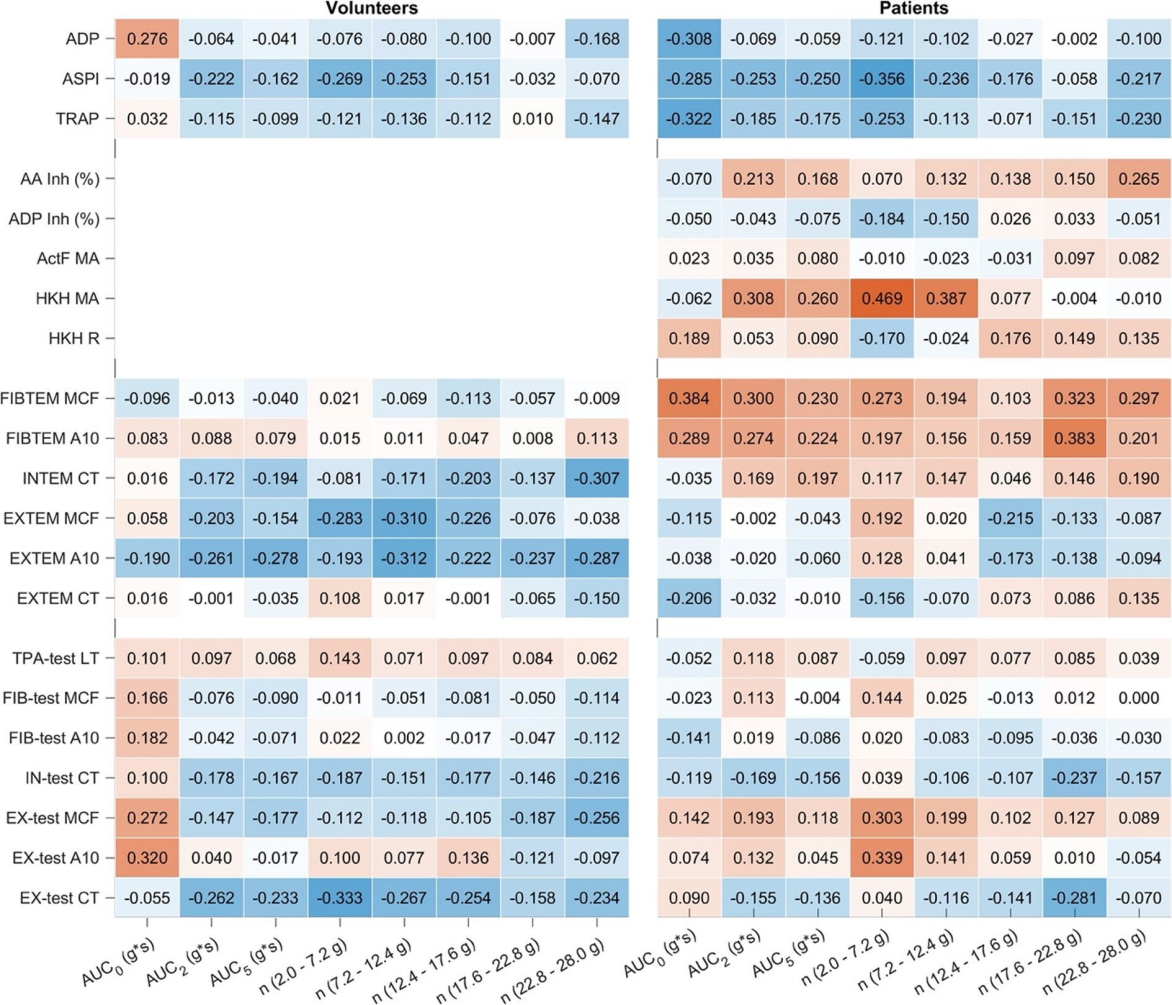
Spearman correlation coefficients (ρ) between acceleration variables and transport-related differences in coagulation and platelet function test results. Each cell represents the correlation between the relative difference (Δ) in a specific test variable (PTS minus manual transport) and a defined acceleration metric. Columns correspond to total AUC or acceleration bin-specific AUCs, grouped by intensity ranges: AUC₀ (≥ 0 g), AUC₂ (≥ 2 g), AUC₅ (≥ 5 g), and acceleration bins (2.0–7.2 g, 7.2–12.4 g, 12.4–17.6 g, 17.6–22.8 g, and 22.8–28.0 g). Left panel: healthy volunteers; right panel: septic patients. The color shading indicates the direction and strength of the correlation (blue = negative, red = positive). Stronger color intensity denotes higher absolute correlation values. No correlation exceeded |ρ| = 0.5, and no coefficient was statistically significant at the *p* < 0.01 level.

**Fig. 5:**
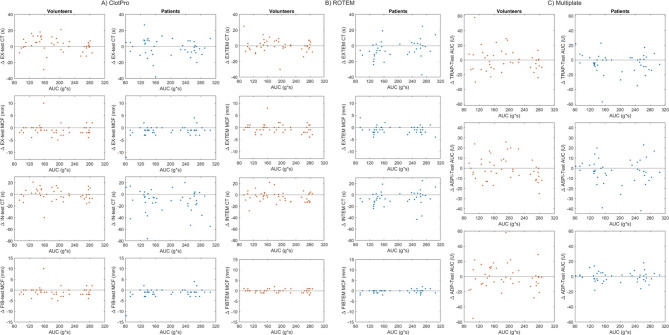
**A-C**. Relationship between transport-induced acceleration forces and changes in viscoelastic and platelet function test results. Scatter plots showing the relationship between the AUC of transport-related acceleration forces and the absolute difference (Δ) in test results between the PTS and manual transport. A: Columns display ClotPro variables in healthy volunteers (left) and septic patients (right), including CT and MCF from the EX-test, CT from the IN-test, and MCF from the FIB-test. B: Columns displaying ROTEM variables in healthy volunteers (left) and septic patients (right), including CT and MCF from EXTEM, CT from INTEM, and MCF from FIBTEM. C: Columns displaying Multiplate platelet aggregation TRAP, ASPI, and ADP tests in healthy volunteers (left) and septic patients (right). Each data point represents an individual measurement pair. No consistent correlation was observed. AUC, area under the curve; PTS, pneumatic tube system; CT, clotting time; MCF, maximum clot firmness; EX-test, extrinsic activation test; IN-test, intrinsic activation test; FIB-test, fibrinogen contribution test; TRAP, thrombin receptor–activating peptide; ASPI, arachidonic acid–induced aggregation; ADP, adenosine diphosphate.

The strength of PTS-induced acceleration, expressed as the AUC, showed consistently weak to moderate correlations with relative variable changes (all Spearman ρ < 0.5, *p* ≥ 0.01; Fig. 4). Figure 5 illustrates the relationship between the AUC of PTS acceleration and the transport-related differences in selected coagulation and platelet function variables. For ClotPro measurements, no clear trend was observed across the AUC range for EX-test CT and MCF, IN-test CT, and FIB-test MCF. Variability of individual data points was comparable across AUC levels in both cohorts. For Multiplate TRAP, ASPI, and ADP tests, differences between PTS and reference samples were distributed without systematic deviation across AUC values. No clustering or dose-dependent patterns were apparent in either healthy volunteers or septic patients.

### Clinical factors affecting PTS-related coagulation changes in septic patients

**Fig. 6:**
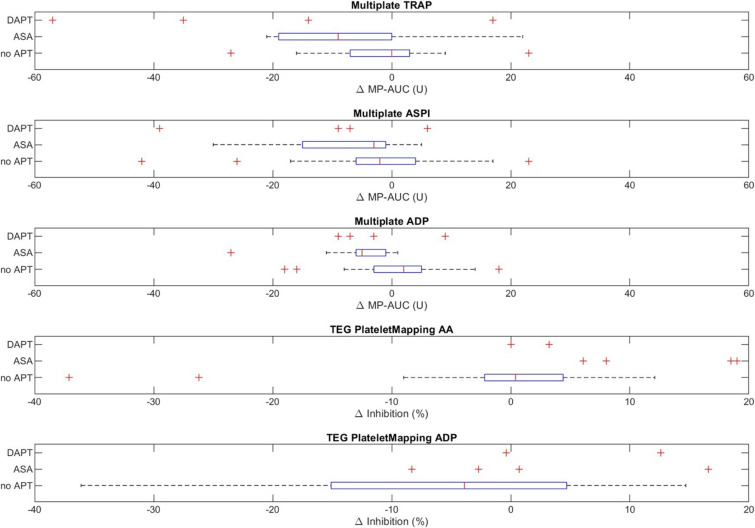
Effects of platelet inhibition on transport-induced changes in platelet aggregation. Boxplots showing the difference in area under the curve (ΔAUC, [U]) between PTS and manual transport for TRAP, ASPI, and ADP Multiplate tests, stratified by platelet inhibition regimen: no antiplatelet therapy, ASA monotherapy, and dual antiplatelet therapy. Negative ΔAUC values indicate reduced platelet aggregation after PTS transport. ΔAUC, absolute difference in area under the curve; TRAP, thrombin receptor–activating peptide; ASPI, arachidonic acid–induced aggregation; ADP, adenosine diphosphate; ASA, acetylsalicylic acid; PTS, pneumatic tube system.

**Fig. 7:**
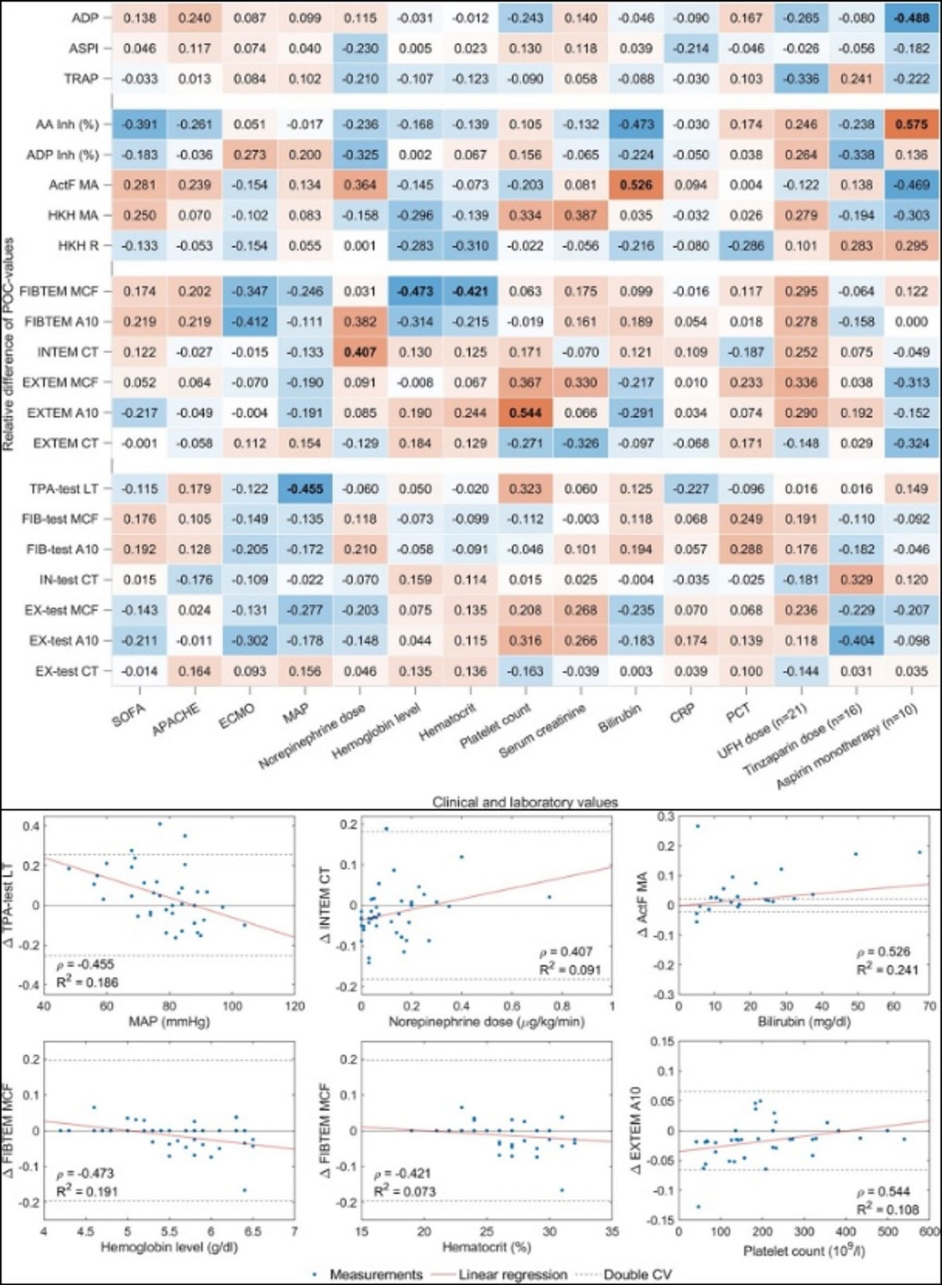
A, **B.** Relationships between relative changes in point-of-care test results after PTS transport and clinical or laboratory variables. **A**: Heatmap showing Spearman correlation coefficients between relative differences in viscoelastic and platelet function test results (PTS vs. manual transport) and selected clinical and laboratory variables. The color intensity indicates the strength and direction of the correlation. Significant associations (*p* < 0.05) are highlighted. **B**: Scatter plots with regression lines and R² values depicting associations between individual clinical or laboratory variables and relative differences in selected viscoelastic or platelet function test results after PTS transport compared with manual handling. The dashed lines represent twice the coefficient of variation (2×CV) threshold for clinically relevant deviation. SOFA, Sequential Organ Failure Assessment; APACHE, Acute Physiology and Chronic Health Evaluation; ECMO, extracorporeal membrane oxygenation; MAP, mean arterial pressure; CRP, C-reactive protein; PCT, procalcitonin; MA, maximum amplitude; R, reaction time; CT, clotting time; A10, amplitude at 10 min; MCF, maximum clot firmness; AA, arachidonic acid; ActF, activated functional fibrinogen test; CV, coefficient of variation.

The impact of antiplatelet therapy on PTS-induced changes in platelet function is shown in Fig. 6; Table 3. Associations between clinical variables and transport-related differences in coagulation test results are illustrated in Fig. 7A. Hemoglobin, hematocrit, platelet count, bilirubin, ASA therapy, and norepinephrine dose significantly influenced the magnitude of these changes. The corresponding regression analyses are shown in Fig. 7B.

**Table 3:** Impact of antiplatelet therapy on PTS-induced change in platelet tests.

	Healthy Volunteers		Septic Patients					
			All		No APT		ASA APT	
	Difference		Difference					
	***Median*** ***(Q25-Q75)***	***p-value***	***Median*** ***(Q25-Q75)***	***p-value***	***Median*** ***(Q25-Q75)***	***p-value***	***Median*** ***(Q25-Q75)***	***p-value***
*Multiplate*			*n* = 45		*n* = 30		*n* = 10	
TRAP [U]	−2 (−10–8)	0.643	−4 (−10–3)	**0.021**	0 (−7–3)	0.121	−9 (−19–0)	0.195
ASPI [U]	−2 (−8–8)	0.955	−3 (−9–3)	0.094	−2 (−7–4)	0.318	−3 (−15 – −1)	0.053
ADP [U]	−2 (−9–11)	0.866	1 (−4–4)	0.743	2 (−3–5)	0.356	−5 (−6 – −1)	**0.016**
*TEG Platelet Mapping*			*n* = 25		*n* = 19		*n* = 4	
ADP Inh [%]	-	-	−2.7 (−9.5–6.7)	0.397	−3.9 (−14.6–4.3)	0.147	−1.0 (−5.5–8.7)	1.0
AA Inh [%]	-	-	2.2 (−0.4–5.8)	0.104	0.1 (−2.6–3.9)	0.879	13.3 (7.0–18.8)	0.125

Table 3: Impact of antiplatelet therapy on PTS-induced changes in platelet function variables. This table presents the change (i.e., the difference) in platelet function test results after PTS transport compared with manual transport. In the septic patient cohort, subgroup analysis was performed based on the presence or absence of antiplatelet therapy with ASA. Data are reported as medians, with the 25th and 75th percentiles, of the difference between PTS and manual transport, with corresponding p-values from the Wilcoxon signed-rank test. PTS: pneumatic tube system; AUC: area under the curve; APT: antiplatelet therapy; ASA: acetylsalicylic acid; TRAP, thrombin receptor-activating peptide; ASPI, arachidonic acid stimulation test; ADP, adenosine diphosphate stimulation test; ADP Inh, adenosine diphosphate inhibition; AA Inh, arachidonic acid inhibition.

### Combined analysis of devices with similar functional profiles

**Fig. 8:**
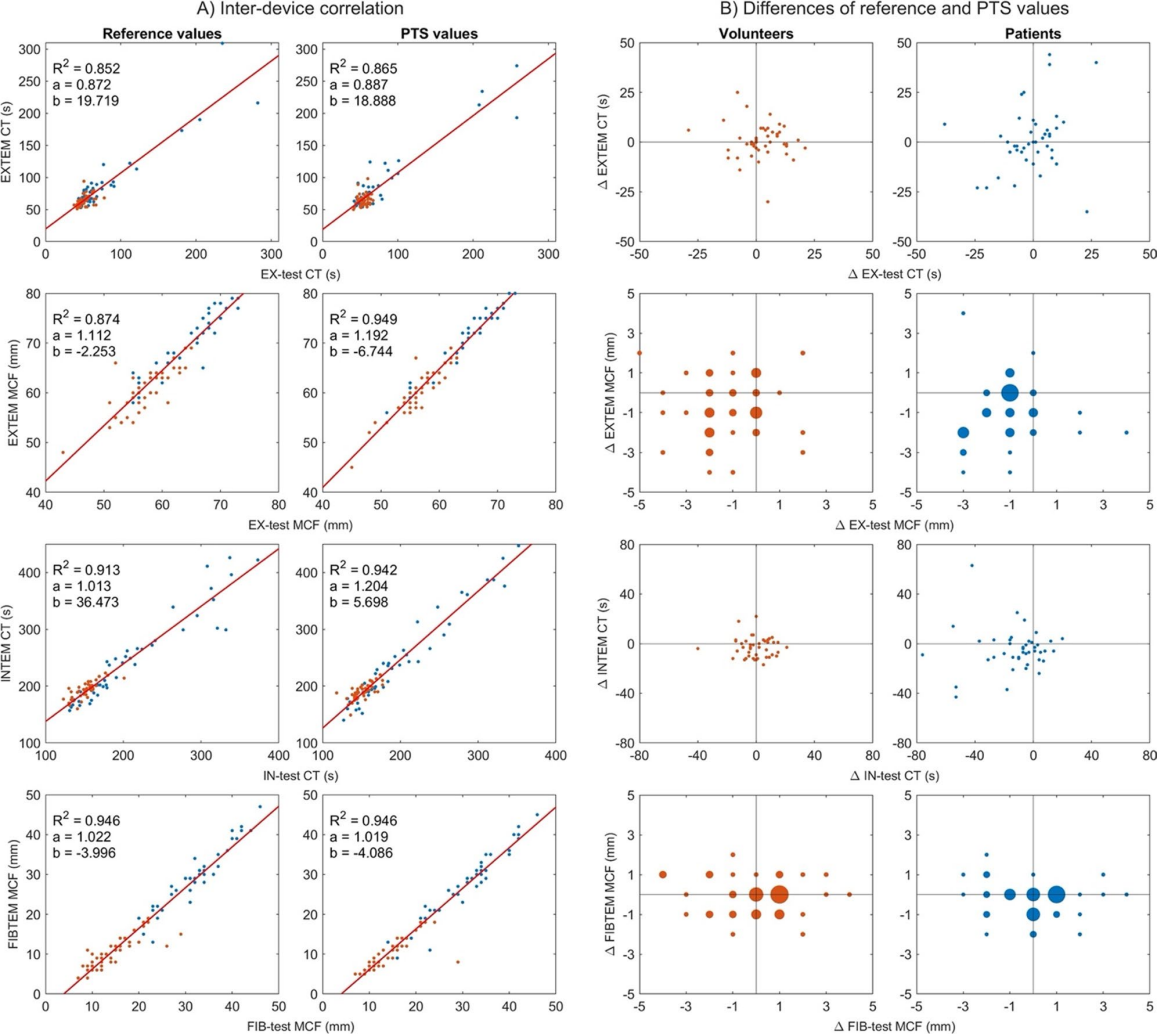
A, **B.** *Correlations and agreements between reference and pneumatic tube system measurements in viscoelastic testing.* **A**: Correlation plots comparing viscoelastic test results (EX-test, IN-test, FIB-test) after manual (reference) and pneumatic tube system (PTS) transport. Linear regression lines and coefficients of determination (R²) are shown. All the comparisons demonstrated strong linear correlation (R² > 0.85). **B**: Bland-Altman-like plots displaying the differences between the reference and PTS results for each variable in healthy volunteers (orange) and septic patients (blue). The bubble size reflects the measurement density. No systematic bias was observed between platforms under either transport condition. PTS, pneumatic tube system; CT, clotting time; MCF, maximum clot firmness; EX-test, extrinsically activated assay; IN-test, intrinsically activated assay; FIB-test, fibrinogen contribution assay; R², coefficient of determination.

The devices ClotPro and ROTEM share similar functional principles in several assays, including EXTEM/EX-test, INTEM/IN-test, and FIBTEM/FIB-test, which typically yield correlated results (Fig. 8A). If acceleration during PTS transport had a systematic impact on measurement outcomes, comparable effects would be expected across both devices. Plotting the transport-induced differences between platforms (Fig. 8B) revealed a symmetrical distribution around the origin for EXTEM/EX-test CT, indicating random variation. In contrast, EXTEM/EX-test MCF differences clustered in the lower left quadrant, particularly in septic patients, suggesting a concordant decrease across both systems. As shown in the analyses above, these changes also remained within the range of clinical equivalence. A similar, although less pronounced, pattern was observed for the INTEM/IN-test CT. No clear clustering was observed for the FIBTEM/FIB-test MCF, consistent with random variation and the absence of a systematic effect.

## Discussion

**Fig. 9:**
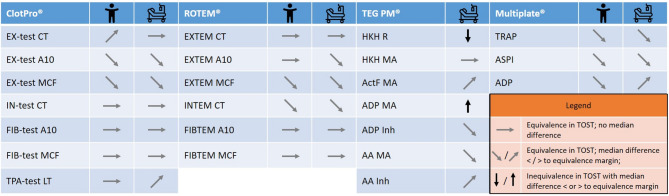
Practical summary of transport effects on viscoelastic and platelet function assays. Paired analyses after manual versus pneumatic tube transport across all analyzers (ClotPro, ROTEM, TEG PlateletMapping, and Multiplate). Each field represents one assay parameter (e.g. CT, A10, MCF, MA, LT). The left column shows results for healthy volunteers, the right column for critically ill patients with sepsis. Color coding denotes equivalence testing (TOST): - Grey: Equivalent results in TOST – observed difference remains within expected intra-device variability; - Black: Not equivalent in TOST – results require cautious interpretation when pneumatic tube transport is used. Arrow direction indicates whether the median difference lies below or above the manufacturer-reported coefficient of variation (CV), reflecting the direction and relative magnitude of deviation.

To our knowledge, this prospective randomized study with sample-level allocation is the first to systematically evaluate the effects of PTS transport on next-generation VET and platelet function testing in healthy individuals and septic patients while quantifying acceleration forces. Across ROTEM, ClotPro, Multiplate, and TEG PlateletMapping, PTS transport did not cause clinically relevant alterations in most variables. As illustrated in Fig. 9, post-transport changes generally remained below manufacturer-reported intra-device variability, confirming the robustness of modern POC coagulation technologies under real-world conditions. Most test results remained within predefined equivalence margins, suggesting that minor deviations reflect intra-device variability rather than transport effects, or cannot be differentiated from them. Robust performance was observed even under high acceleration, though TEG PlateletMapping – especially the HKH R-variable and ADP/AA inhibition – requires cautious interpretation.

These findings contrast with earlier reports suggesting preanalytical vulnerability of coagulation testing^16,28,29^. Prior studies often lacked direct force measurement, used outdated platforms, or included only healthy subjects, limiting clinical relevance^16,17,28–31^. The VETaPT trial combined modern devices, three-axis acceleration measurement, and a septic cohort, improving methodological accuracy. Logistic regression showed no dose-dependent association between AUC and viscoelastic CT deviation. Samples with CT shifts ≥ 10 s had even lower median AUC, indicating that biological and analytical noise rather than mechanical stress drives observed variability and that that transport-related effects are not dose-dependent within the investigated range. Consequently, analysis focused on direct comparison of transport conditions. Equivalence testing was used to determine whether these differences remained within clinically acceptable limits. Both septic and healthy cohorts showed comparable resilience. Though IN-test CT in septic patients shortened by 7 s after PTS, equivalence testing confirmed clinical acceptability within a ± 16 s margin. Trends toward shorter CT after PTS were consistent with previous reports, though absolute differences remained below clinical thresholds. Two studies reported a an IN-test CT shortening of about 13 s following PTS transport^16,30^, with another showing a similar trend^28^. Likewise, one study observed a comparable R-time reduction in the HKH test using the TEG 5000 system despite methodological differences^17^. Earlier studies describing platelet inhibition after transport lacked acceleration data and reported high intrinsic device variability. Two studies reported a pronounced reduction in Multiplate results after PTS transport, which was not reflected in our data^29,32^. Neither study included direct quantification of acceleration forces. Another investigation assessing PTS effects on Multiplate reported high baseline interdevice variability – 16% for the TRAP test and 26% for the ASPI test – even without transport, with variability further increasing after PTS^33^. The underlying cause of reduced measurable platelet function following PTS transport remains unclear across studies. The hypothesis of platelet activation and exhaustion remains speculative.

Methodological strengths include equivalence testing using Wilcoxon-based TOST and bootstrap method, transparent reporting of margins, and clinically interpretable outcomes. For most assays, observed changes did not exceed manufacturer-reported intra-device variation, confirming the robustness of modern POC systems. Limitations include single-center design, potential underpowering for rare variables, and restriction to anticoagulated septic patients. Allocation was performed at the sample level using a non-systematic approach. In addition, blinding was incomplete, as the investigator performing ClotPro and TEG PlateletMapping measurements was aware of transport allocation. While this may introduce observer-related bias, all assays followed standardized procedures with largely automated readouts, reducing the likelihood of a clinically relevant influence on the results. The use of CV-based equivalence margins has limitations. While this approach ensures that transport-related differences remain within the analytical imprecision of the devices, it does not exclude the presence of small systematic effects. The analytical strategy evolved following the absence of a detectable AUC-response relationship. While equivalence margins were defined a priori, the application of equivalence testing in this context should be interpreted accordingly. Such effects may consistently shift measurements in one direction and become detectable when aggregating multiple samples. Although these shifts would remain indistinguishable from analytical variability at the level of individual measurements, they could theoretically influence clinical interpretation at the population level. Hemolysis was not systematically assessed using objective measures such as a hemolysis index. Although no macroscopic hemolysis or device-related errors were observed, subclinical hemolysis cannot be excluded and may represent an unmeasured preanalytical factor. Missing data were handled using device-specific complete-case analysis. Missingness was primarily related to technical factors; however, as the underlying mechanism was not formally tested, a missing completely at random assumption cannot be confirmed. Although temperature during transport was not actively controlled, comparable draw-to-analysis times and equally handling of paired samples reduce the likelihood of systematic preanalytical bias between transport modalities.

Nonetheless, controlled variation of transport intensity captured the range likely present in clinical environments. Clinically, PTS transport of whole blood for VET and platelet function testing is safe and reliable, even in sepsis-associated coagulopathy. Only TEG PlateletMapping HKH R and ADP/AA inhibition-variables warrant caution. Although TEG PlateletMapping has been shown to predict clinical outcomes in peripheral arterial disease^34^, its correlation with other platelet function assays remains inconsistent. In addition, methodological factors such as temperature, particularly in stored platelet concentrates, have been reported to influence measurement results^35^. Published study also demonstrated poor agreement between TEG PlateletMapping and Multiplate in patients receiving ASA^36^. This may indicate that ASA therapy could have contributed to the observed variability in our cohort, although the current evidence remains inconclusive. However, TEG PlateletMapping has shown good performance in detecting the effects of ADP receptor antagonists^37^.

## Conclusions

Next-generation VET and platelet function tests are largely resistant to mechanical stress from PTS transport. Minor deviations fall within intra-device variability. Only selected TEG PlateletMapping variables exhibited variability, indicating limited robustness. The VETaPT trial supports the safe integration of PTS into diagnostic workflows for most VET devices. Future research should examine high-risk contexts such as trauma or ECMO.

## Supplementary Information

Below is the link to the electronic supplementary material.

Supplementary material 1 (DOCX 403.2 kb)

## Data Availability

The datasets generated and analyzed during the current study will be available in the Open Access Repository and Archive for Research Data of Saxon Universities OPARA (https://opara.zih.tu-dresden.de/home) (38). For further inquiries, the corresponding author can be contacted.

## Abbreviations

A10: Amplitude at 10 min after clotting time
AA: Arachidonic acid
ActF: Activator F (used in TEG PlateletMapping) test
aPTT: Activated partial thromboplastin Time
ASA: Acetylsalicylic acid (Aspirin)
ASPI: Aspirin test (Multiplate assay for platelet function)
AUC: Area under the curve (used for acceleration force quantification)
BE: Blood extraction
BMBF: Federal ministry of education and research (Germany)
CFT: Clot formation time
CI: Confidence Interval
CRP: C-Reactive Protein
CT: Clotting Time
FIB-test: Fibrinogen test (ClotPro)
FIBTEM: ROTEM-based fibrinogen assay
HKH: Heparinized kaolin-activated heparinase assay (TEG assay)
INR: International normalized ratio
IN-test: Intrinsic pathway test (ClotPro)
INTEM: Intrinsic ROTEM test
LT: Lysis time
MCF: Maximum clot firmness
MA: Maximum amplitude (in TEG)
MAP: Mean arterial pressure
LMWH: Low-molecular-weight heparin
PCT: Procalcitonin
POC: Point-of-Care
PRP: Platelet-rich plasma
PTS: Pneumatic tube system
Q25/Q75: 25th / 75th Percentile (Interquartile Range)
ROTEM: Rotational Thromboelastometry
SAPS II: Simplified Acute Physiology Score II
SD: Standard Deviation
SEPSIS-3: Third international consensus definition for sepsis
SLT: Standard Laboratory Test
SOFA: Sequential organ failure assessment
TOST: Two one-sided test (statistical equivalence testing)
TRAP: Thrombin receptor activating peptide test (Multiplate)
UFH: Unfractionated heparin
VET: Viscoelastic testing
