# Supplementary material for: Effects of pneumatic tube systems on next-generation viscoelastic coagulation test devices in septic patients and healthy individuals: Results of the randomized controlled VETaPT trial
Source: Sci Rep. 2026 May 28;16:16587. doi: 10.1038/s41598-026-54938-7 (PMC13219619; doi:10.1038/s41598-026-54938-7)
Supplement: Supplementary file 1 — Supplementary material 1 (DOCX 403.2 kb) [file 41598_2026_54938_MOESM1_ESM.docx]

*Table S1: Acceleration forces during different types of transport within the VETaPT trial.*

|  | *AUC [g*s]* | *Median Count of Acceleration Events* | | | | |
| --- | --- | --- | --- | --- | --- | --- |
|  | *Median*  *(Q25-Q75)* | *Acceleration bin in g-range* | | | | |
|  |  | *2.0-7.2g* | *7.2-12.4g* | *12.4-17.6g* | *17.6-22.8g* | *22.8-28.0g* |
| *Transport mode* |  |  |  |  |  |  |
| Manual | 5  (1–12) | 161 | 0 | 0 | 0 | 0 |
| PTS | 185  (138–263) | 1361  (1188–1655) | 159  (119–254) | 68  (50–76) | 56  (35–76) | 33  (16–60) |
| *PTS Modifications* |  |  |  |  |  |  |
| Slow + bag | 119  (99–132) | 1087  (952–1156) | 86  (75–107) | 42  (25–51) | 31  (21–47) | 22  (12–26) |
| Fast + bag | 225  (191–250) | 1367  (1251–1495) | 175  (156–200) | 91  (66–104) | 83  (64–109) | 69  (45–98) |
| Slow + insert | 148  (139–158) | 1328  (1217–1442) | 132  (120–160) | 53  (50–61) | 37  (34–42) | 13  (11–20) |
| Fast + insert | 271  (262–278) | 1738  (1671–1789) | 293  (281–303) | 118  (112–123) | 74  (69–82) | 59  (54–64) |

Table S1: The table presents the acceleration forces measured during the VETaPT study across different transport modes. For PTS transport, acceleration forces are shown for all PTS transports combined, as well as those stratified by transport modifications used to modify speed. Acceleration was quantified using the following variables: AUC, calculated by integrating the recorded acceleration values; the number of acceleration events within predefined acceleration clusters (bins); transport velocity. Where appropriate, values are reported as medians with the 25th and 75th percentiles (Q25 and Q75). PTS: pneumatic tube system. AUC: area under the curve.


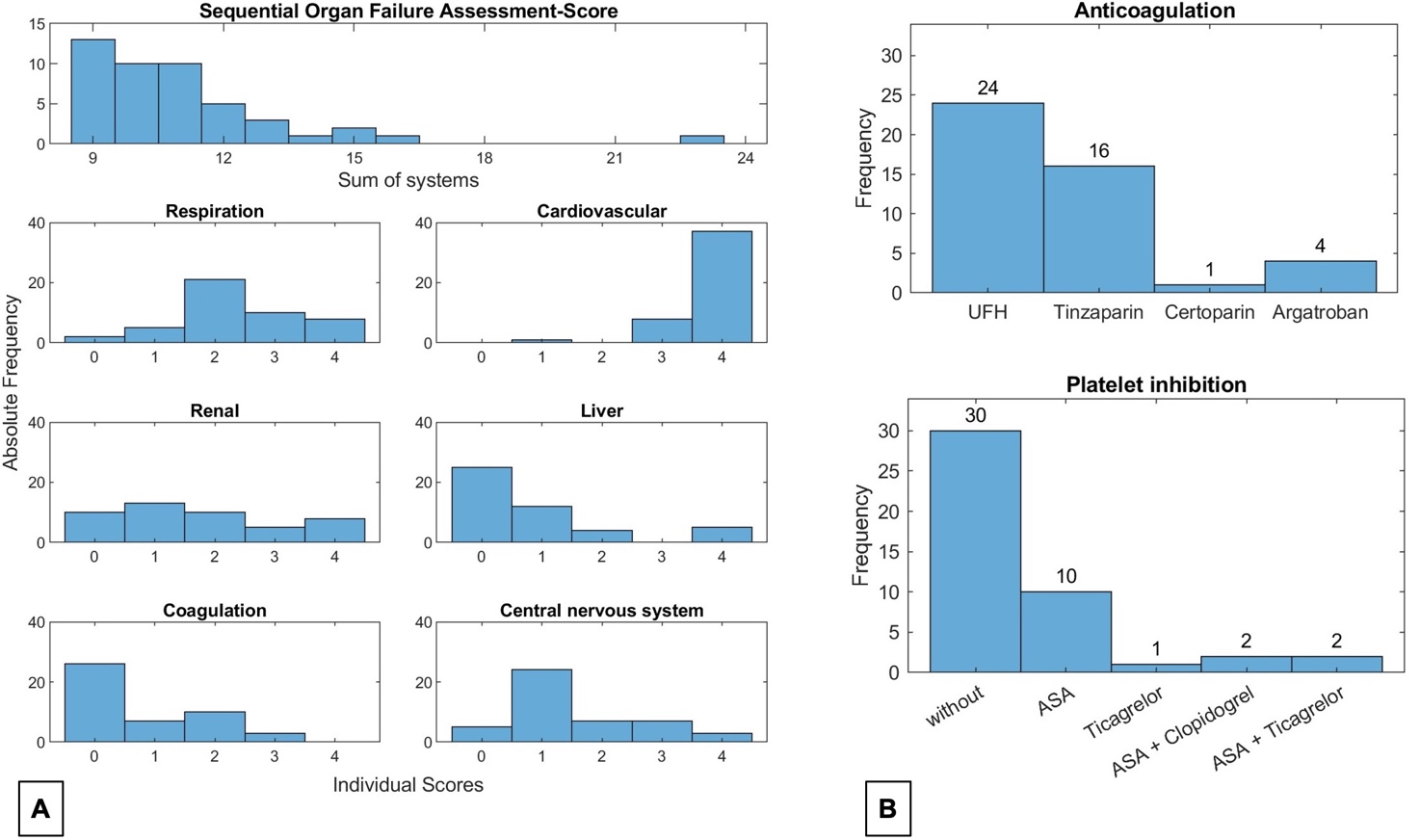


Figure S1A, B. Clinical characteristics of the septic patients included in the study. A: SOFA score and its individual organ-specific components at the time of study inclusion. B: Frequencies of anticoagulant and antiplatelet therapies administered to septic patients at the time of sampling. SOFA, Sequential Organ Failure Assessment; LMWH, low molecular weight heparin; UFH, unfractionated heparin; ASA, acetylsalicylic acid.


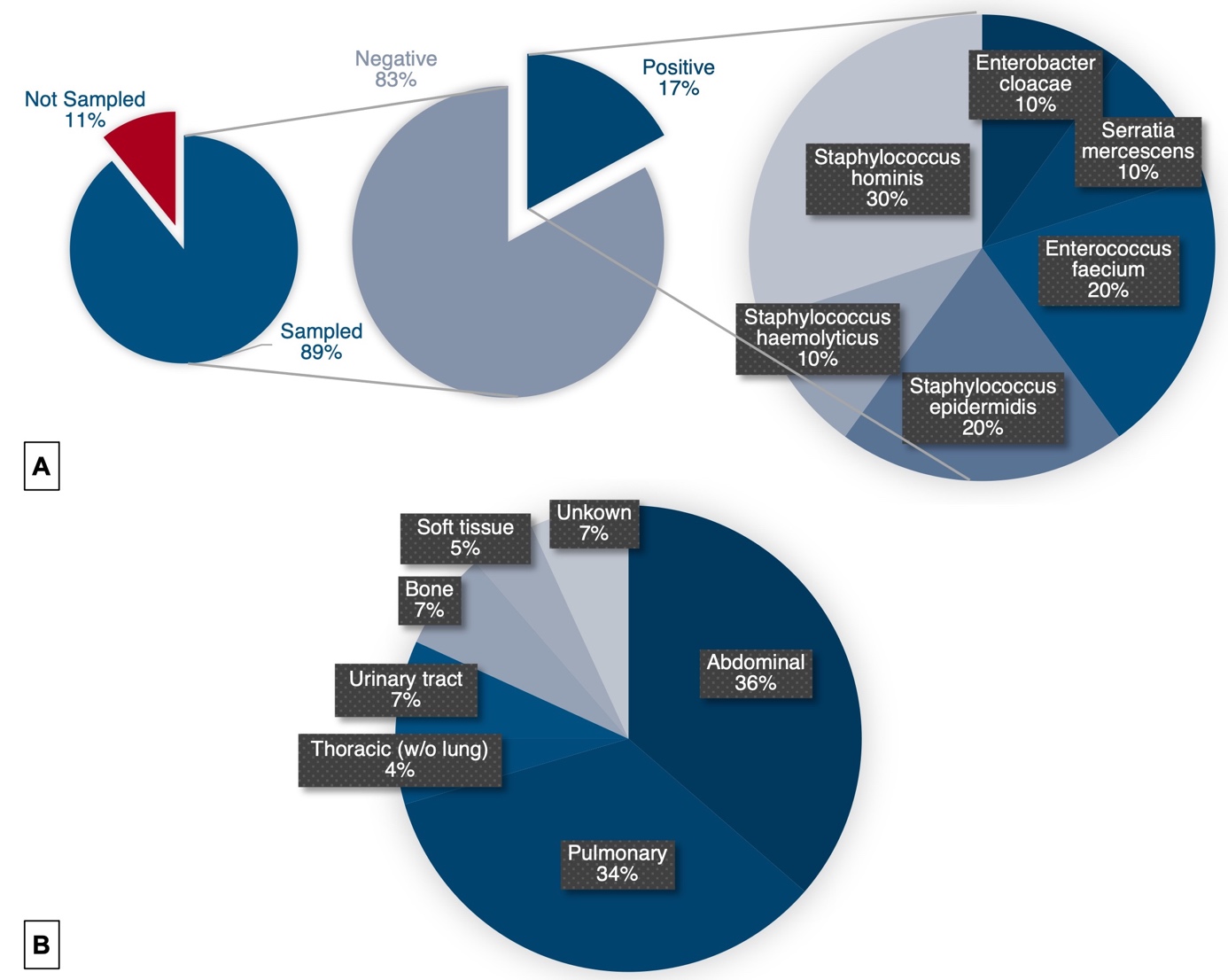


Figure S2A, B. Blood culture results and presumed focus in septic patients included in the

study.

A: Results of blood culture diagnostics performed on the day before, the day of, or the day after inclusion. The proportion of positive cultures, and the identified pathogens are shown. B: The presumed clinical source of sepsis is displayed. CT, clotting time; A10, amplitude at 10 min; R, reaction time; MCF, maximum clot firmness; MA, maximum amplitude; LT, lysis time; ADP, adenosine diphosphate; ASPI, arachidonic acid–induced aggregation; TRAP, thrombin receptor–activating peptide; AA Inh, arachidonic acid % inhibition; ADP Inh, ADP % inhibition; ActF MA, activated functional fibrinogen maximum amplitude; HKH, heparinized kaolin-activated heparinase assay; EX-test, extrinsic activation assay; IN-test, intrinsic activation assay; FIB-test, fibrinogen contribution assay; TOST, two one-sided tests for equivalence; CV, coefficient of variation.
